# Non-persistence with anti-platelet therapy and long-term mortality after ischemic stroke: A nationwide study

**DOI:** 10.1371/journal.pone.0244718

**Published:** 2021-02-09

**Authors:** Seung Jae Kim, Oh Deog Kwon, Ho Chun Choi, Eung-Joon Lee, BeLong Cho

**Affiliations:** 1 Department of Family Medicine, Seoul St. Mary’s Hospital, College of Medicine, The Catholic University of Korea, Seoul, Republic of Korea; 2 Republic of Korea Navy 2nd Fleet Medical Corps, Pyeongtaek-si, Gyeonggi-do, Republic of Korea; 3 Department of Family Medicine, Healthcare System Gangnam Center, Seoul National University Hospital, Seoul, Republic of Korea; 4 Nuvizen, Palo Alto, CA, United States of America; 5 Department of Neurology, Seoul National University Hospital, Seoul, Republic of Korea; 6 Department of Family Medicine, Seoul National University Hospital, Seoul, Republic of Korea; Medizinische Universitat Graz, AUSTRIA

## Abstract

**Background:**

We tried to investigate the effect of non-persistence with antiplatelets after ischemic stroke on long-term all-cause mortality (ACM).

**Methods and findings:**

We selected newly diagnosed ischemic stroke patients aged ≥20years who were newly treated with aspirin or clopidogrel from 2003–2010 Korean National Health Insurance Service-National Sample Cohort, a random sample of 2.2% of total population. Subjects were divided into two pairs of groups according to persistence with antiplatelets at 6 and 12 months: those who discontinued antiplatelets within 6 months (DA6M) and those who continued them for 6 or months or more (CA6M); and those who discontinued antiplatelets within 12 months (DA12M) and those who continued them for 12 months or more (CA12M). Those who died within 6 months among DA6M and those who died within 12 months among DA12M were excluded along with those with medication possession ratio<80% among CA6M and CA12M. Subjects were followed-up until death or December 31, 2013. Among 3,559 total subjects, DA6M were 1,080 and CA6M were 2,479 while, out of 3,628 total patients, DA12M were 1,434 and CA12M were 2,194. The risks of ACM [adjusted hazard ratio (aHR), 2.25; 95% confidence interval (CI), 1.94–2.61], cerebro-cardiovascular disease (CVD) death (aHR, 2.52; 95% CI, 1.96–3.24) and non-CVD death (aHR, 2.11; 95% CI, 1.76–2.64) of DA6M were all significantly increased compared to CA6M. DA12M also had significantly higher risks of ACM (aHR, 1.93; 95% CI, 1.65–2.25), CVD mortality (aHR, 2.13; 95% CI; 1.63–2.77) and non-CVD mortality (aHR, 1.83;95% CI 1.51–2.22) than DA12M but aHRs were lower than that between DA6M and CA6M. The difference rates of ACM, CVD death, and non-CVD death between non-persistent and persistent groups all continuously widened over time but the degree of difference was gradually decreased.

**Conclusions:**

Maintaining antiplatelets for the first 12 months after ischemic stroke reduces long-term risks of both CVD death and non-CVD death.

## Introduction

Globally, stroke is one of the leading causes for death and also long-term disability [1]. Of these strokes, ischemic stroke accounts for approximately 87% [2] and many ischemic stroke survivors experience serious vascular complications including death over the course of years [3, 4].

Previous meta-analyses of randomized trials have proved antiplatelet therapy as a foundation to prevent recurrent stroke, myocardial infraction (MI), and vascular death in patients with prior history of ischemic stroke [5]. Furthermore, American Heart Association/American Stroke Association (AHA/ASA) guidelines recommend that antiplatelet therapy need to start with drugs such as aspirin or clopidogrel for secondary prevention of stroke in patients with ischemic stroke [6–8]. Nevertheless, non-persistence with antiplatelet medications is known to be pandemic and this was associated with poor clinical outcomes including increased mortality for patients with cardiovascular diseases [9]. The results of the Prospective Registry Evaluating Myocardial Infarction: Event and Recovery (PREMIER) study showed that hazard ratio of one year mortality for patients with acute MI who discontinued aspirin after one month to those who continued aspirin was 1.82 (95% CI: 1.09–3.03) [10]. Another study reported that 16% of patients who received drug eluting stent implantation did not fill clopidogrel prescriptions on the day of discharge and they had higher rates of death or MI (14.2% versus 7.9%, p<0.001) compared to patients who filled clopidogrel on discharge [11].

Meanwhile, in case of post-ischemic stroke patients, persistence with antiplatelet agents was also reported to decline rapidly during the first 1–2 years after ischemic stroke [12, 13], and non-persistence was related with increased risk of early recurrent stroke, MI and vascular death [14, 15]. However, the observation period of previous studies was relatively short and there were no data regarding premature discontinuation of antiplatelets on all-cause death. Thus, the aim of this study was to examine the effect of non-persistence with antiplatelet medications on the long- term all-cause mortality for patients with newly diagnosed ischemic stroke in a real- world setting by using a large-scale representative National claims data of Korea.

## Materials and methods

### Data source

The data set for this nationwide cohort study was acquired from the National Health Insurance Service-National Sample Cohort (NHIS-NSC) of Korea. NHIS-NSC is a population-based cohort created by the National Health Insurance Service (NHIS) of Korea in 2002. All Koreans are mandatorily enrolled to the NHIS as either Medicare beneficiaries (97%) or Medicaid beneficiaries (3%), hence NHIS provides universal healthcare coverage to all Korean citizens. NHIS-NSC is a randomly extracted cohort sample population of 1,017,468 individuals, which is approximately 2.2% of the entire Korean population (N = 46,605,433). The NHIS-NSC is obtained annually through continuous observation of the data. This includes qualification data (age, sex, household income, type of health insurance, residential area, death record etc.), medical service claims data (diagnosis record, healthcare service record, billing statement etc.), and pharmacy claims data (Name of drugs, prescription date, number of supplied days of drugs, dosage & frequency of drugs etc.) [16]. The exemplary data of NHIS-NSC has been well established [16, 17]. Our study was approved by the institutional review board (IRB) at the Seoul National University Hospital (IRB No.E-15-5-079-673) and the written consent from each patient was waived since the NHIS-NSC data were fully anonymized according to the strict confidentiality guidelines. This study was also approved by National Health Insurance review committee (NHIS-2017-2-610).

### Study population

**Fig 1:**
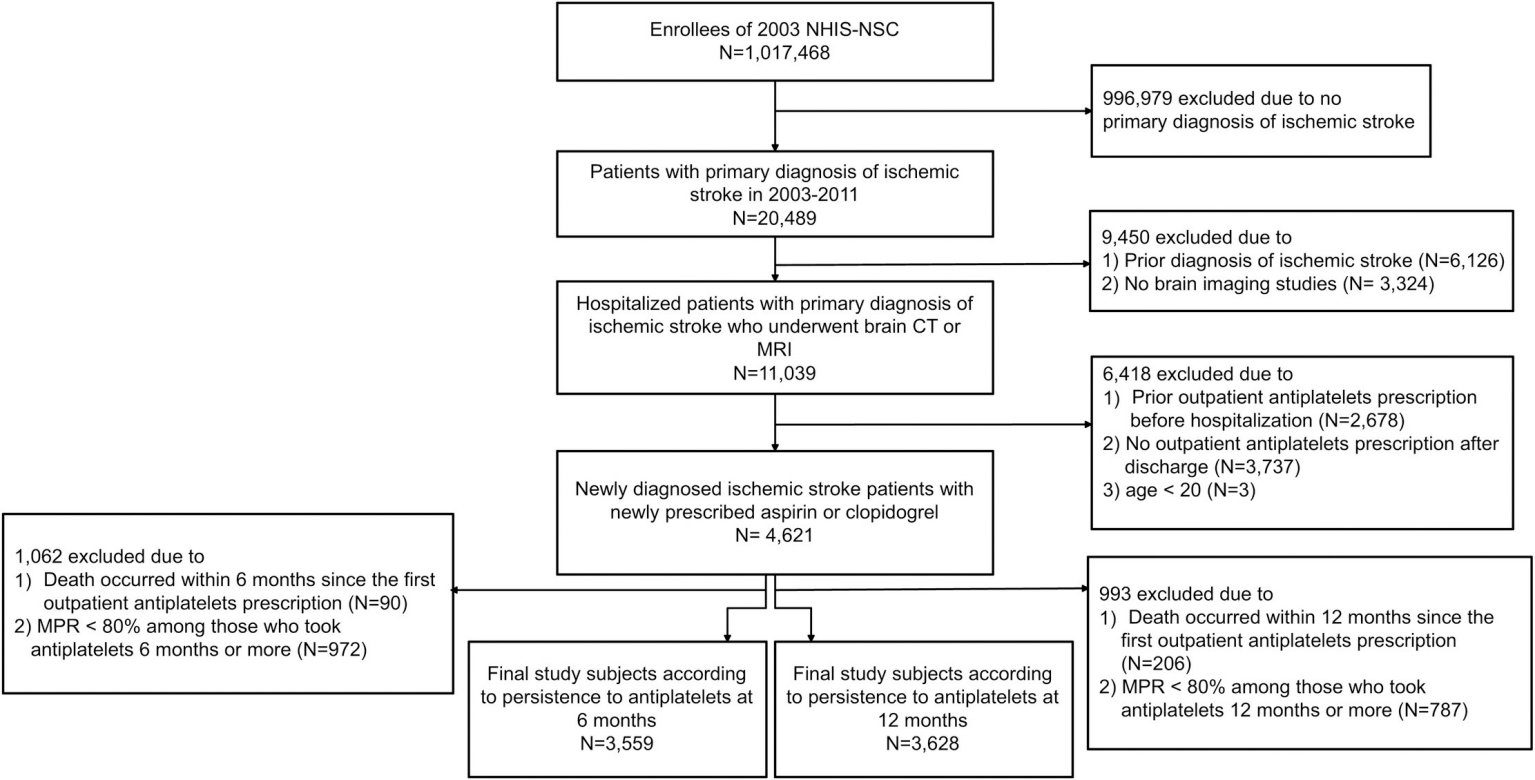
Study population and data collection. NHIS-NSC, National Health Insurance Service-National Sample Cohort; CT, computed tomography; MRI, Magnetic resonance imaging; MPR, Medication possession ratio.

We selected newly diagnosed ischemic stroke patients aged 20 years or older, who newly started antiplatelet therapy between years 2003 to 2010 (N = 20,489). Ischemic stroke patients were defined as hospitalized subjects with a primary diagnosis of ischemic stroke (International Classification of Disease, 10^th^ revision, ICD-10: I63, I64, I65, I66) who underwent either brain computed tomography (CT) or magnetic resonance imaging (MRI) during hospitalization (N = 11,039). The reason for limiting subjects with brain imaging record was due to the assumption that the ischemic stroke patients need to undergo brain imaging studies [18], and previous studies that selected post-stroke patients from NHIS-NSC also have adapted this definition [19, 20]. The subjects with prior records of ischemic stroke (I63-I66) diagnosis or outpatient antiplatelet prescription before the index hospitalization dates were excluded to include only newly diagnosed ischemic stroke patients who were newly treated with antiplatelets. Antiplatelet agents that were included in this study were limited to aspirin and clopidogrel the most commonly prescribed antiplatelet agents to patients with ischemic stroke [6, 7, 21]. We did not include other antiplatelet agents besides aspirin or clopidogrel since they were seldom used because Korean health insurance coverage was not applied to them at the time of the observation period. Patients with no outpatient prescription of aspirin or clopidogrel after discharge were also excluded. Thus, patients who were newly treated with aspirin or clopidogrel were selected according to the Anatomical Therapeutic Chemical (ATC) classification code [22] (N = 4621). In order to figure out the effect of non-persistence with antiplatelets on long-term mortality, we performed the survival analysis between those who discontinued antiplatelets within 6 months since the first prescription (DA6M) and those who continued to take them for the next 6 months or more (CA6M). Further, to evaluate the impact of longer persistence with antiplatelets on the mortality in newly diagnosed post-ischemic stroke patients, we also conducted additional analysis between those who discontinued antiplatelets within 12 months since the first prescription (DA12M) and those who continued to take them for the next 12 months or more (CA12M). Subjects who died within 6 months since the first antiplatelet prescription were excluded for the analysis between the DA6M and CA6M groups while patients who died within 12 months were excluded for the analysis between the DA12M and CA12M groups. Moreover, adherence to antiplatelets of CA6M and CA12M patients were calculated using medication possession ratio (MPR), defined as the total days of medication supplied divided by the number of days between the first and last prescription refill [23] and those with poor adherence (MPR < 80%) to antiplatelets were excluded. A MPR of 80% has been well-established as a reasonable cutoff point when determining good or poor medication adherence using claims data [23, 24] hence, it is also widely used to define medication persistence [25, 26]. Among total cohort subjects, 3559 eligible subjects were finally selected for analysis between the DA6M and CA6M groups and 3628 were chosen for analysis between the DA12M and CA12M groups based on the inclusion criteria mentioned above (Fig 1).

### Study outcome

The primary outcome of this study was all-cause mortality and secondary outcomes were cerebro-cardiovascular disease (CVD) mortality and non-CVD mortality. CVD mortality was defined as death cases due to cerebrovascular disease (ischemic and hemorrhagic stroke, I60-69) and ischemic heart disease (I20-25) while the rest was considered as non-CVD death. The cause and date of death were attained from death record from the qualification data of NHIS-NSC. The patients were followed-up from the first outpatient prescription date of antiplatelets after ischemic stroke to the occurrence of death or until the end of observation period (December 31, 2013).

### Definition of medication non-persistence

The outpatient prescription of antiplatelets of each patient was observed for 2–3 years since the first prescription. We defined medication non-persistence (discontinuation) as only when the prescription of antiplatelets was discontinued and was not refilled for the remainder of the observed prescription period. Patients who continued to take aspirin or clopidogrel since the first prescription of antiplatelet agents were considered as continuers regardless of drug switch to one another. If a patient was on both aspirin and clopidogrel, they were regarded as discontinuers only when both were discontinued.

### Statistical analysis

We applied time-dependent Cox proportional model to calculate the hazard ratios of study outcomes (all-cause mortality, CVD death and non-CVD death) between the DA6M and CA6M groups, and DA12M and CA12M groups. Hazard ratios were adjusted for age, sex, income, residential area, type of health insurance, and the Charlson Comorbidity Index (CCI). The CCI score was calculated before the start of observation period using ICD-10 codes [27]. The proportion of Kaplan-Meier estimator was used to demonstrate survival plot. All statistical analyses were performed using STATA version 14.1 (Stata Corp., College Station, TX, USA) and a two-sided p value < 0.05 was defined as statistically significant.

## Results

### Baseline characteristics

There were 3,559 newly diagnosed ischemic stroke patients who were newly treated with aspirin or clopidogrel when analyzed according to the persistence with antiplatelets at 6 months. Among them, 30.3% (N = 1,080) of patients discontinued antiplatelets prematurely within 6 months and 56.3% were male, while 43.7% were female. Mean age of the total subjects was 66.1±12.1 years and 70.3% of patients were 60 years or older. Majority of subjects (84.9%) resided in a metropolitan area or a city and 73.4% were in the upper-middle income class. Out of the total subjects, 93.3% were Medicare beneficiaries and 6.7% were benefiting from Medical aid. Regarding the comorbidities, 72.7% of subjects had CCI scores less than 4 and 27.3% had 4 or higher. The duration of antiplatelets for DA6M averaged 49.3±56.7 days while CA6M took them for 732.1±228.0 days in average (Table 1).

**Table 1:** Characteristics of subjects according to persistence with antiplatelets at 6 months.

Characteristics	All n (%) or mean±SD	< 6 months n (%) or mean±SD	6 ≥ months n (%) or mean±SD	p value
Total	3559 (100.0)	1080 (100.0)	(100.0) 2479	
Sex				0.198
Male	2002 (56.3)	590 (54.6)	1412 (57.0)	
Female	1557 (43.7)	490 (45.4)	1067 (43.0)	
Age (year)	66.1±12.1	67.5±12.3	65.5±11.9	0.000
20–49	361 (10.1)	100 (9.3)	261 (10.5)	
50–59	697 (19.6)	171 (15.8)	526 (21.2)	
60–69	1007 (28.3)	305 (28.2)	702 (28.3)	
70–79	1094 (30.7)	336 (31.1)	758 (30.6)	
≥ 80	400 (11.3)	168 (15.6)	232 (9.4)	
Income				0.001
Low	949 (26.6)	324 (30.0)	625 (25.2)	
Middle	1116 (31.4)	351 (32.5)	765 (30.9)	
High	1494 (42.0)	405 (37.5)	1089 (43.9)	
Residence				0.003
Metropolitan area	1461 (41.1)	411 (38.0)	1050 (42.4)	
City	1561 (43.8)	476 (44.1)	1085 (43.7)	
Rural	537 (15.1)	193 (17.9)	344 (13.9)	
Health insurance				0.003
Medicare	3320 (93.3)	987 (91.4)	2333 (94.1)	

Abbreviation: SD, standard deviation.

Meanwhile, a total of 3,628 subjects were selected for our analysis according to the persistence with antiplatelets at 12 months. In this case, the prevalence of premature discontinuation of antiplatelets within 12 months was 39.5% (N = 1,434). Among them, 55.8% were male and 44.2% were female. Mean age was 65.9±12.1 years and 69.7% were 70 years or older. The upper-middle income class covered 73.5% and 84.6% were residents of metropolitan area or city. Among them, 93.3% were beneficiaries of Medicare and 72.9% had CCI score less than four. The average duration of antiplatelets for DA12M and CA12M were 116.4±116.3 and 792.7±162.5 days, respectively (Table 2).

**Table 2:** Characteristics of subjects according to persistence with antiplatelets at 12 months.

Characteristics	All n (%) or mean±SD	< 12 months n (%) or mean±SD	12 ≥ months n (%) or mean±SD	p value
Total	3628 (100.0)	1434 (100.0)	2194 (100.0)	
Sex				0.024
Male	2024 (55.8)	767 (53.5)	1257 (57.3)	
Female	1604 (44.2)	667 (46.5)	937 (42.7)	
Age (year)	65.9 ± 12.1	66.7 ± 12.6	65.4 ± 11.7	0.000
20-49	379 (10.5)	149 (10.5)	228 (10.4)	
50-59	719 (19.8)	246 (17.2)	473 (21.6)	
60-69	1028 (28.3)	402 (28.0)	626 (28.5)	
70-79	1114 (30.7)	434 (30.3)	680 (31.0)	
≥ 80	388 (10.7)	201 (14.0)	187 (8.5)	
Income				0.000
Low	961 (26.5)	418 (29.1)	543 (24.7)	
Middle	1151 (31.7)	471 (32.9)	680 (31.0)	
High	1516 (41.8)	545 (38.0)	971 (44.3)	
Residence				0.000
Metropolitan	1482 (40.8)	559 (39.0)	923 (42.1)	
City	1588 (43.8)	609 (42.5)	979 (44.6)	
Rural	558 (15.4)	266 (18.5)	292 (13.3)	
Health insurance				0.001
Medicare	3384 (93.3)	1314 (91.6)	2070 (94.3)	
Medical aid	244 (6.7)	120 (8.4)	124 (5.7)	
Charlson comorbidity index				0.048
< 4	2646 (72.9)	1020 (71.1)	1626 (74.1)	
≥ 4	982 (27.1)	414 (28.9)	568 (25.9)	
Average duration of antiplatelets	525.4±361.5	116.4±116.3	792.7±162.5	0.000

Abbreviation: SD, standard deviation.

There was a tendency that non-persistent group (DA6M and DA12M) were slightly older, had more rural residents, Medical aid beneficiaries and CCI scores higher than 4 but less income. The proportion of women were also higher in non-persistent groups compared to persistent groups but statistically significant trend was only found in DA12M group (Tables 1 and 2).

### Mortality according to the persistence with antiplatelet medication

We compared the long-term mortality between DA6M (N = 1,080) and CA6M (N = 2,479) and also between DA12M (N = 1,434) and CA12M (N = 2,194). Median follow-up duration for analysis between DA6M and CA6M was 4.28 years while it was 4.38 years for analysis between DA12M and CA12M. In outcome analysis between DA6M and CA6M, there were total of 356 cases of all-cause death (132 CVD deaths and 224 non-CVD deaths) for DA6M group. The CA6M group also had 356 total all-cause deaths with 118 CVD deaths and 238 non-CVD deaths specifically. The risks of all-cause mortality, CVD-mortality, and non-CVD mortality were all significantly increased for the DA6M group compared to the CA6M group with adjusted hazard ratio (HR) of 2.25 (95% CI, 1.94–2.61; p = 0.000), 2.52 (95% CI 1.96–3.24, p = 0.000), 2.11 (95% CI, 1.76–2.64; p = 0.000) respectively. In outcome analysis between the DA12M and CA12M groups, there were 381 total cases of all-cause death (137 CVD deaths and 244 non-CVD deaths) in the DA12M group, while 288 all-cause deaths (95 CVD deaths and 193 non-CVD deaths) have occurred in the CA12M group. The DA12M group also had a higher risk of all-cause death, CVD death, and non-CVD death than CA12M group with adjusted HRs of 1.93 (95% CI; 1.65–2.25, p = 0.000), 2.13 (95% CI, 1.63–2.77; p = 0.00) and 1.83 (95% CI, 1.51–2.22; p = 0.00), respectively. These results were all statistically significant (Table 3).

**Table 3:** Non-persistence with antiplatelets and long-term mortality after ischemic stroke.

**Duration of antiplatelets**	All-cause mortality					CVD mortality					Non-CVD mortality				
	Event No	Crude HR	p value	Adjusted HR^a^	p value	Event No	Crude HR	p value	Adjusted HR^a^	p value	Event No	Crude HR	p value	Adjusted HR^a^	p value
< 6 months vs ≥ 6 months^b^															
< 6 months (n=1080)	356	2.53 (2.19-2.93)	0.000	2.25 (1.94-2.61)	0.000	132	2.83 (2.21-3.63)	0.000	2.52 (1.96-3.24)	0.000	224	2.38 (1.98-2.56)	0.000	2.11 (1.76-2.54)	0.000
≥ 6 months (n=2479)	356	Reference		Reference		118	Reference		Reference		238	Reference		Reference	
< 12 months vs. ≥ 12 months^c^															
< 12 months (n=1434)	381	2.10 (1.80-2.44)	0.000	1.93 (1.65-2.25)	0.000	137	2.29 (1.76-2.98)	0.000	2.13 (1.63-2.77)	0.000	244	2.00 (1.66-2.42)	0.000	1.83 (1.51-2.22)	0.000
≥12 months (n=2194)	288	Reference		Reference		95	Reference		Reference		193	Reference		Reference	

Abbreviation: CVD, cerebro-cardiovascular disease; non-CVD, non-cereboro-cardiovascular disease; HR, hazard ratio.

Analyses were performed using time dependant Cox proportional model.

^a^Adjusted for age, sex, income, residential area, type of insurance, and Charlson Comorbidity Index

^b^Median follow-up was 4.28 years

^c^Median follow-up was 4.38 years

### Kaplan-Meier survival curves

**Fig 2:**
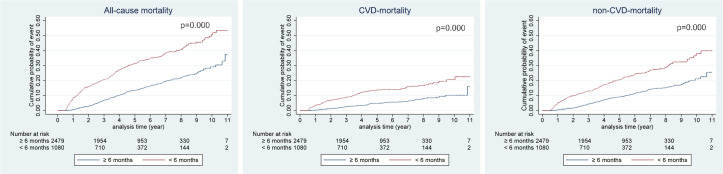
Kaplan-Meier curves of cumulative probability of event according to persistent with antiplatelets at 6 months after ischemic stroke. CVD, cerebro-cardiovascular disease; non-CVD, non-cerebro-cardiovascular disease.

**Fig 3:**
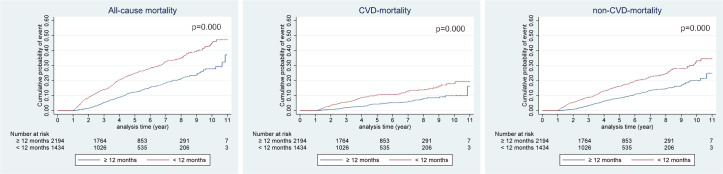
Kaplan-Meier curves of cumulative probability of event according to persistent with antiplatelets at 12 months after ischemic stroke. CVD, cerebro-cardiovascular disease; non-CVD, non-cerebro-cardiovascular disease.

The Kaplan-Meier curve for cumulative probability of event between each DA6M and CA6M groups demonstrated that the difference rates of all-cause mortality, CVD mortality and non-CVD mortality all continuously increased further for the rest of the observation period while the degree of increase gradually decreased over time. This trend was remained to be seen on the survival curve of all outcomes between DA12M and CA12M groups but with less degree of difference (Figs 2 and 3).

## Discussion

The results of our study demonstrated high prevalence of premature discontinuation of antiplatelets for ischemic stroke survivors in a real-world setting. Approximately 30% of patients discontinued antiplatelets prematurely within the first 6 months after the initiation and almost 40% of the patients discontinued them within a year. These findings were consistent with previous studies where non-persistence rates of antiplatelets after ischemic stroke were 30–45% [15, 28].

Our study also found an association between non-persistent with antiplatelet therapy and increased risk of long-term all-cause mortality among newly diagnosed ischemic stroke patients who started antiplatelets for the first time. Patients who prematurely discontinued antiplatelets demonstrated significantly higher rates of both CVD death and non-CVD death than those who persistently took antiplatelets. Higher CVD death rate for non-persistent group is likely due to higher incidences of recurrent stroke or ischemic heart diseases such as MI for them which lead to more CVD deaths. The increased risk of recurrence for ischemic stroke survivors are well known [29, 30] as the actuarial risk of recurrence within a year after the initial stroke is reported to be 13%, which is around 15 times the risk compared to general population [31]. There are also increased risk of MI or other coronary artery diseases (CAD) after ischemic stroke since they share common risk factors and pathological mechanisms [32, 33]. According to the recent Canadian large population-based cohort study, ischemic stroke had independently increased risk of major adverse cardiovascular events such as acute coronary syndrome, acute MI, incident CAD, and so on, regardless of sex [34]. These risks are tend to increase even more when patients discontinue antiplatelets prematurely [14, 15] and we believe this has substantially contributed to increased risk of long-term CVD mortality for them.

In case of greater risk of non-CVD mortality for non-persistent groups compared to persistent groups, it could be primarily due to more frequent recurrent strokes and coronary artery diseases for them which provoke increased risk of various complications [35]. Longer hospitalizations, functional and cognitive impairments in consequences of more recurrent strokes, and other cardiovascular events for the non-persistent groups could have resulted more non-vascular deaths due to events such as falls, aspiration pneumonia, pulmonary embolism, nosocomial infections, depression, dementia, and so on [35]. Therefore, the proper initiation and maintenance of antiplatelet therapy after the ischemic stroke should be highly emphasized in accordance with the guidelines [8].

Interestingly, despite the statistically significant increase in mortality risk that was confirmed in our analysis between DA12M and CA12M groups, its hazard ratio was found to be lower than that between DA6M and CA6M groups. Furthermore, we also additionally performed the survivor analysis between DA6M and CA12M and it demonstrated the highest adjusted hazard ratios for all-cause death (aHR 2.49, 95% CI 2.13–2.91), CVD death (aHR 2.81, 95% CI 2.15–3.67), and non-CVD death (aHR 2.32, 95% CI 1.91–2.83). Thus, this study may have provided the real-world evidence that maintaining antiplatelets during the first 6 months after the initial stroke may be even more important. Further real-world analysis aiming to determine the optimal duration of antiplatelets beyond 12 months, which brings significantly positive effect on the long- term mortality risk for stroke survivors, should be performed in the near future.

Furthermore, Kaplan-Meier curves for cumulative probability of event between the non-persistent and persistent groups illustrated that the difference rates of all-cause mortality, CVD mortality, and non-CVD mortality all persistently widened further over the course of the observation period though the degree of difference gradually decreased. This trend could be explained by a “health adherer effect” [36]. Patients who are adherent to medications are more likely to own other healthy behaviors such as not smoking and exercising regularly and vice versa. Thus, there is a strong possibility that those who prematurely discontinued antiplatelets have unhealthy lifestyle and they could be non-adherent to other prescribed drugs as well. Non-adherence to other cardiovascular medications, such as statins and anti-hypertensive drugs attributes to 40–80% increased risk of cardiovascular events according to the meta-analysis performed by Chowdhury et al [37]. We believe that these factors have resulted a continuously increased risk of mortality throughout the observation period for patients who were non-persistent to antiplatelets during the first 12 months after ischemic stroke.

This study has several strengths. First, our study well reflects the real-world situation by examining the data of every ischemic stroke patient included in a large-scale, representative nationwide population-based cohort database. We also excluded subjects who had previous diagnoses of ischemic stroke or antiplatelet prescriptions before the index date to limit potential bias. Moreover, we adjusted various confounding factors such as age, income, residential area, type of insurance and comorbidities which could affect the medication persistence and adherence through qualification and medical service claims data of NHIS-NSC. Lastly, we observed the death events of subjects for maximum of 11 years. To our knowledge, our study is the first to investigate the association between the premature discontinuation of antiplatelet therapy after ischemic stroke and the long-term mortality risk up to 11 years. In addition, no previous study has reported risks of non-CVD death and all-cause death due to non-persistence with antiplatelet drugs after ischemic stroke especially using a large-scale claims data.

However, our results should be interpreted in light of some potential limitations. First, we could not distinguish the severity and specific types of ischemic stroke since we were not able to confirm the findings of imaging studies due to the nature of claims data. Second, we could not include variables regarding lifestyle factors such as smoking and alcohol consumption since they were not available in the NHIS-NSC. However, we could suppose that these factors were presumably distributed evenly to each comparison groups considering the fact that subjects were continuously extracted for a quite a long period of time and the number of selected samples were relatively sufficient. Third, we did not reflect the adherence status of other secondary preventive medications (e.g. antihypertensive, antidiabetic and lipid lowering agents) that could also influence the risk of mortality in the analysis. Even though this information was available in the NHIS-NSC, analyzing persistence and adherence of these medications by tracking every single prescription of various drugs is a very difficult task which requires highly complicated operational definition given the fact that the any one of these drugs could be changed, added or discontinued during the any point of study period. Thus, we settled to exclude them as covariates and assumed that the antiplatelet non-persistent group would also likely to be non-adherent to other secondary preventive drugs based on the “health adhere effect. Fourth, since we only examined the persistence with antiplatelets, we may have mis-categorized patients who switched from antiplatelets to anticoagulant due to detection of paroxysmal atrial fibrillation (pAF) after discharge into non-persistent group. However, the range and extent of diagnostic work-up to detect pAF for cryptogenic stroke varies greatly from center to center and follow-up electrocardiogram (ECG) monitoring are not performed in majority of cases after discharge [38]. Furthermore, detection rates of pAF were only 8.9% of cryptogenic stroke patients at 6 months and 12.4% at 12 months even with an insertable cardiac monitor and only 1.4% at 6 months and 2.0% at 12 months with conventional ECG monitoring [39]. Thus, the percentage of patients who switched to anticoagulants due to newly detected pAF in this study is believed to be quite low. Moreover, warfarin was only available anticoagulant during the study period. Thus, even if we did find all the patients who switched to warfarin, it would have been technically impossible to assess the persistent and adherent status of warfarin correctly because its dose and prescriptions are adjusted according to patients’ time in therapeutic range. In addition, the mortality risk for non-persistent group in this study was significantly higher even with the possible inclusion of persistent patients who switched to warfarin in the non-persistent group. Thus, we believe that the impact of this limitation on the overall results of our study would be less significant. Fifth, we also could not include use of over-the-counter (OTC) drugs in the analysis since NHIS-NSC only contains information of prescribed drugs. Aspirin is attainable through both the prescription and OTC in Korea. However, prescribed aspirins can be obtained at a discounted price via Korean health insurance coverage compared to OTC aspirins [40]. Therefore, we could assume that most patients would take a dose of aspirin with a prescription rather than OTC purchase. In addition, previous study also indicated missed OTC exposure may not invalidate results based on prescription data [41]. Sixth, claim-based measurement of persistence and adherence has some flaws as well. Patients might have obtained medications from different sources (e.g. drug samples, drug sharing, etc.) and filling a prescription does not necessarily indicate ingestion of medication [42, 43]. However, measuring adherence through claims data is still considered to be objective and it provides more accurate illustration of real-world medication use than clinical trials [43, 44]. Finally, despite the use of well standardized study populations, our study subjects were limited to only Koreans. Thus, our findings may not be generalized to other racial or ethnical groups and additional studies featuring multi-ethnic populations would be needed.

## Conclusions

In this retrospective cohort study, premature discontinuation of antiplatelets within 6 or 12 months after ischemic stroke was quite common and this was significantly associated with increased risks for both the CVD mortality and non-CVD mortality over time. Thus, physicians should make constant efforts to encourage and educate patients to be persistent and adherent with antiplatelets after ischemic stroke, especially for the first 12 months.
