# Correction: Non-persistence with anti-platelet therapy and long-term mortality after ischemic stroke: A nationwide study

**DOI:** 10.1371/journal.pone.0314217

**Published:** 2024-11-14

**Authors:** The PLOS One Staff

The first two authors, Seung Jae Kim and Oh Deog Kwon, should be noted as contributing equally to this work.

In Table 1, the rows Medicaid under Health Insurance, Charlson comorbidity index and Average duration of antiplatelets are missing. Please see the correct Table 1 here.

**Table 1:** Characteristics of subjects according to persistence with antiplatelets at 6 months.

Characteristics	All n (%) or mean±SD	< 6 months n (%) or mean±SD	6 ≥ months n (%) or mean±SD	p value
Total	3559 (100.0)	1080 (100.0)	2479 (100.0)	
Sex				0.198
Male	2002 (56.3)	590 (54.6)	1412 (57.0)	
Female	1557 (43.7)	490 (45.4)	1067 (43.0)	
Age (year)	66.1±12.1	67.5±12.3	65.5±11.9	0.000
20–49	361 (10.1)	100 (9.3)	261 (10.5)	
50–59	697 (19.6)	171 (15.8)	526 (21.2)	
60–69	1007 (28.3)	305 (28.2)	702 (28.3)	
70–79	1094 (30.7)	336 (31.1)	758 (30.6)	
≥ 80	400 (11.3)	168 (15.6)	232 (9.4)	
Income				0.001
Low	949 (26.6)	324 (30.0)	625 (25.2)	
Middle	1116 (31.4)	351 (32.5)	765 (30.9)	
High	1494 (42.0)	405 (37.5)	1089 (43.9)	
Residence				0.003
Metropolitan area	1461 (41.1)	411 (38.0)	1050 (42.4)	
City	1561 (43.8)	476 (44.1)	1085 (43.7)	
Rural	537 (15.1)	193 (17.9)	344 (13.9)	
Health insurance				0.003
Medicare	3320 (93.3)	987 (91.4)	2333 (94.1)	
Medicaid	239 (6.7)	93 (8.6)	146 (5.9)	
Charlson comorbidity index				0.006
< 4	2586 (72.7)	751 (69.5)	1835 (74.0)	
≥ 4	973 (27.3)	329 (30.5)	644 (26.0)	
Average duration of antiplatelets	524.9±368.5	49.3±56.7	732.1±228.0	0.000

The publisher apologizes for the errors.
